# Electrochemical Characterization of Modified Glassy Carbon Electrodes for Non-Enzymatic Glucose Sensors

**DOI:** 10.3390/s21237928

**Published:** 2021-11-27

**Authors:** Julia Maria Mazurków, Anna Kusior, Marta Radecka

**Affiliations:** Faculty of Materials Science and Ceramics, AGH University of Science and Technology, 30-059 Kraków, Poland; akusior@agh.edu.pl (A.K.); radecka@agh.edu.pl (M.R.)

**Keywords:** non-enzymatic sensors, glucose sensors, copper sulfides, diffusion-controlled process, electrochemical impedance spectroscopy, IHOAM model

## Abstract

The diversity of materials proposed for non-enzymatic glucose detection and the lack of standardized protocols for assessing sensor performance have caused considerable confusion in the field. Therefore, methods for pre-evaluation of working electrodes, which will enable their conscious design, are currently intensively sought. Our approach involved comprehensive morphologic and structural characterization of copper sulfides as well as drop-casted suspensions based on three different polymers—cationic chitosan, anionic Nafion, and nonionic polyvinylpyrrolidone (PVP). For this purpose, scanning electron microscopy (SEM), X-ray diffraction (XRD), and Raman spectroscopy were applied. Subsequently, comparative studies of electrochemical properties of bare glassy carbon electrode (GCE), polymer- and copper sulfides/polymer-modified GCEs were performed using electrochemical impedance spectroscopy (EIS) and voltammetry. The results from EIS provided an explanation for the enhanced analytical performance of Cu-PVP/GCE over chitosan- and Nafion-based electrodes. Moreover, it was found that the pH of the electrolyte significantly affects the electrocatalytic behavior of copper sulfides, indicating the importance of OH_ads_ in the detection mechanism. Additionally, diffusion was denoted as a limiting step in the irreversible electrooxidation process that occurs in the proposed system.

## 1. Introduction

The most practical and thus commercialized methods for the self-monitoring of diabetes are based on the electrochemical measurement of glucose levels. Classical sensing setups utilize enzymes, glucose oxidase, or glucose dehydrogenase. The first generation of enzymatic sensors is based on the measurement of hydrogen peroxide produced during enzyme-catalyzed glucose oxidation. However, the dependence on oxygen presence in the system severely hinders their reliability, which has become an incentive for the development of the next generation in which a mediator (e.g., quinones) is responsible for electron transfer. Here, the major drawback is the interference effect of other electroactive species. A real breakthrough in point-of-care diagnosis was the proposal of direct electron transfer implemented by connecting electrically active enzyme sites with the electrode [1,2]. It also triggered the emergence of a new path in bioactive molecule detection, which acquired the status of the fourth sensor generation. In this case, enzymes are substituted by nanomaterials, which are directly involved in glucose oxidation [3,4]. This approach also offers the possibility to bypass some intrinsic limitations of enzymes, such as vulnerability to detergents and sterilization, demanding immobilization procedures, and activity changes due to temperature, pH, and humidity variations [1].

Non-enzymatic glucose oxidation has been the subject of intensive studies for almost 40 years. The first stage of research, dating back to 1982, concerned understanding the nature of glucose electrochemistry on platinum electrodes [5]. However, because of the very sluggish reaction kinetics of noble metal electrodes, unsatisfactory sensitivity values were obtained. Other problems included the interference effect of adsorbed intermediates and the lack of selectivity [1]. To date, various approaches have been proposed to overcome these issues and bring enzyme-less sensors to practical applications by enabling the electrocatalytic mechanism of detection. This involves the development of electrodes based on alloys, bimetallics, carbon, and metal compounds [6]. However, despite the very promising reports of non-enzymatic sensors with extraordinary analytical performance, the basic understanding of the mechanism behind it is missing. It is of prime importance, especially in view of the increasing demand for reliable glucose monitoring systems working in biological fluids other than blood, such as sweat or interstitial fluid.

The mechanism of glucose non-enzymatic electrooxidation has been widely investigated in recent years; however, it remains an active area of research. Hitherto, two major theories have been proposed to explain this issue. The first one, the activated chemisorption model suggested by Pletcher, underlines the role of transition metal unfilled d-orbitals, which participate in the adsorption process. Moreover, it was suggested that hydrogen abstraction from glucose occurs simultaneously with molecule adsorption [7]. This description explains the significance of the geometric factors but fails to consider an enhanced electrocatalytic activity of noble metals in an alkaline medium. This issue was addressed by the IHOAM (Incipient Hydrous Oxide-Adatom Mediator) model. Burke proposed that surface atoms (adatoms) undergo oxidation at low voltages and incipient hydrous oxide premonolayer (OH_ads_) forms. It mediates glucose oxidation, but at the same time, inhibits reduction processes [8]. Nonetheless, this model applies only to noble metals and their oxides. In the case of transition metals, such as copper or nickel, it was later established that the applied potential triggers their oxidation (acquiring a higher oxidation state) than OH_ads_ formation. The oxidized metal itself bonds OH_ads_ radicals, which in turn, are involved in glucose electrooxidation [9,10]. Moreover, metal sulfides remain a very promising group with analytical properties not yet fully explored.

A reliable assessment of the modified electrodes’ sensing capabilities requires an in-depth understanding of the processes occurring at the electrolyte-material and material-electrode interfaces. This is especially challenging for nanomaterials, which can exhibit distinctive properties depending on their shape, size, and surface development [11]. One of the most powerful tools for evaluating the characteristics of an electrochemical system is electrochemical impedance spectroscopy (EIS). This technique offers the possibility of studying the state of the electrode during various processes that occur in electrochemical experiments, such as adsorption, charge, and mass transport [12,13,14,15]. Moreover, the changes introduced by the modification of the substrate electrode with various conductive or nonconductive materials can be assessed using this technique [15,16,17,18,19,20,21,22]. Another field of application of EIS is the direct investigation of the sensors’ analytical response [15,16,17,18,19]. Nonetheless, to fully exploit the potential of this technique, it remains crucial to ensure a reliable interpretation of obtained data and to relate them to the electrode sensing properties [19,23].

In our previous article, we investigated particle-binder interactions in drop-casted suspensions and their influence on the distribution of copper sulfides on the electrode surface [24]. The distinctive sensing properties of electrodes based on the same material, but immobilized using different polymers, were explained by variations in the arrangement of particles in dry deposits. In this study, the focus was shifted to track the changes in the electrochemical characteristics of the modified electrodes introduced by each component. The determined equivalent circuit parameters, as well as the heterogenic rate constants of the electrode reactions, served as a basis for the interpretation of the analytical performance of the modified electrodes. The lowest charge transfer resistance and the highest heterogenic rate constant denoted the copper sulfides/PVP-modified GCE as the most suitable for sensing purposes. Moreover, it was found that alkaline conditions trigger the electrocatalytic behavior of copper sulfides, however, only to some extent.

## 2. Materials and Methods

### 2.1. Reagents and Materials

Low molecular weight chitosan and phosphate-buffered saline (PBS, BioUltra) were purchased from Sigma-Aldrich (Saint Louis, USA). Anhydrous copper chloride (CuCl_2_), thiourea (CH_4_N_2_S), anhydrous ethanol (C_2_H_5_OH), anhydrous glucose (C_6_H_12_O_6_), potassium chloride (KCl), potassium ferricyanide (K_3_[Fe(CN)_6_]), potassium ferrocyanide (K_4_[Fe(CN)_6_]), acetic acid (CH_3_COOH, 99.5–99.9%), sulfuric acid (H_2_SO_4_, 95%), and sodium hydroxide solution (NaOH, 0.1 M) were obtained from Avantor (Gliwice, Poland). Polyvinylpyrrolidone (PVP, M.W. 40,000) and Nafion D-520 dispersion (5 wt.%) were supplied by Alfa Aesar (Haverhill, USA). The chemicals were analytical grade and were used without additional purification. Aqueous solutions of 1 M glucose, 25% acetic acid, 0.1 M KCl + 0.2 mM [Fe(CN)_6_]^3−/4−^, and 0.1 M KCl + 1 mM [Fe(CN)_6_]^3−^ were prepared with deionized water. Silver chloride electrodes (Ag/AgCl, 3 M KCl), platinum wire (diameter of 0.7 mm), and glassy carbon electrodes (GCE, diameter of 3 mm) were manufactured and provided by Mineral Company (Warszawa, Poland). The glassy carbon used in the GCEs was produced during the two-step pyrolysis of highly cross-linked furan-based epoxy resin at a maximum temperature of 2200 °C (type G).

### 2.2. Preparation and Characterization of Copper Sulfides

Copper sulfide microstructures were obtained according to a previously reported procedure [25]. In the synthesis, 62.5 and 112.5 mM solutions of CuCl_2_ and thiourea, respectively, were prepared in 160 mL of ethanol. Then, 1 g of PVP was added. The solution was mixed on the magnetic stirrer and placed in a Teflon-lined stainless-steel autoclave (maximum capacity of 300 mL). The synthesis temperature was set to 200 °C and time to 6 h. Afterward, the autoclave was cooled to room temperature naturally. The obtained product was washed with a water/ethanol solution (1:1 volume ratio), centrifuged, and dried in a vacuum oven at 60 °C for 12 h.

Scanning electron microscopy (SEM) was applied for the investigation of copper sulfide morphology. Images were obtained using a Nova NanoSEM 200 (FEI Company, Hillsboro, USA). The structure was determined by Raman spectroscopy performed on Raman confocal microscope alpha 300R (WITec GmbH, Ulm, Germany). Spectra were collected in the range of 200–1600 cm^−1^ at 488 nm excitation wavelength. For the evaluation of the phase composition, X-ray diffraction (XRD) was utilized. Analysis was conducted by X’Pert MPD diffractometer (Malvern Panalytical Ltd., Malvern, UK) equipped with Johansson monochromator (Cu Kα_1_ radiation, 1.5406 Å). The specific surface area of the powder was measured using the ASAP 2020 Plus analyzer (Micrometrics, Norcross, GA, USA).

### 2.3. Preparation of Modified Electrodes

GCEs were modified with copper sulfides using different polymers as binders (chitosan, Nafion, and PVP), as described in detail in our previous work [24]. Firstly, pre-treatment of bare GCEs was performed to ensure their reproducibility. For this purpose, the GCEs surface was polished with alumina slurry (0.3 µm), cleaned in water and ethanol, and electrochemically activated in 0.1 M H_2_SO_4_ following the procedure proposed by Gao et al. [26]. The next step involved the preparation of three suspensions containing an appropriate amount of binder and copper sulfides denoted Cu-CH (chitosan-based), Cu-NAF (Nafion-based), and Cu-PVP (PVP-based). As a solvent, 25% acetic acid was used for Cu-CH and Cu-PVP suspensions. In the case of Nafion, the commercially available solution (water/1-propanol) was diluted with distilled water in a 1:9 ratio. To ensure homogeneity, 30 min of magnetic stirring and 10 min of ultrasounds were applied. Subsequently, 10 µL of as-prepared suspensions were casted on GCEs and left to dry in air for 24 h. When not in use, modified electrodes were stored in the refrigerator, suspended over the deionized water. For comparison purposes, GCEs modified with suspensions containing only the binders (CH—chitosan, NAF—Nafion) were also prepared. The quantitative composition of all suspensions is summarized in Table 1.

### 2.4. Electrochemical Characterization of Modified Electrodes

Modified glassy carbon electrodes were applied as working electrodes for measurements using electrochemical impedance spectroscopy (EIS). The tests were conducted in a two-electrode system with a Pt wire as a counter electrode. Impedance spectra were obtained by means of a Solartron 1260 response frequency analyzer with a 1296 dielectric interface (Solartron Analytical, Armstrong Mall, UK). The frequency range of 10^−2^–10^6^ Hz and AC signal amplitude of 10 mV was applied. Analysis of the measured spectra was performed with ZView software (AMETEK Scientific Instruments, Berwyn, IL, USA). In the model used to fit the experimental spectra, the unit of the circuit consisted of resistors (R), constant phase elements (CPE), and Warburg element (W).

All voltammetry experiments were conducted using an electrochemical analyzer M161E (MTM Anko, Kraków, Poland) in a three-electrode system with bare or modified GCE serving as a working electrode, silver chloride electrode (Ag/AgCl, 3 M KCl) as a reference electrode, and platinum wire as an auxiliary electrode. To determine the electrochemically active surface area of the electrodes, the cyclic voltammetry (CV) technique was applied. As an electrolyte, a solution of 0.1 M KCl + 1 mM [Fe(CN)_6_]^3−^ was used. The voltammograms were recorded at different scan rates (6.25–500 mV/s) in the potential range −100–1000 mV.

### 2.5. Glucose Detection

To evaluate the glucose sensing performance of modified electrodes, cyclic voltammetry and chronoamperometry (CA) measurements were conducted. Electrodes were mounted in a cell with a Teflon cup containing 15 mL of an electrolyte. The glucose oxidation potential was evaluated based on CV measurements in 0.1 M NaOH (pH = 13) applied as an electrolyte. The potential range and scan rate were selected as −100–1000 mV and 100 mV/s, respectively. The dependence of the electrode signal on the electrolyte pH was investigated by collecting voltammograms in 0.1 M PBS (pH = 7) and diluted 0.1 M NaOH solutions with adjusted pH values of 13, 12, 11, and 10. CA experiments in 0.1 M NaOH (pH = 13) served to prepare calibration curves and determine the linear range. The blank signal was recorded for 3 min and the signal for subsequent glucose additions for 1 min. A constant potential was chosen based on the oxidation peak potential from CV (1.5 mM glucose addition). Before and after each CV or CA measurement, electrodes were washed with deionized water. Additionally, before recording the proper voltammograms or the amperometric response, 10 initial scans were performed in 0.1 M NaOH (potential range −100–1000 mV).

## 3. Results

### 3.1. Characterization of Copper Sulfides and Drop-Casted Layers

**Figure 1:**
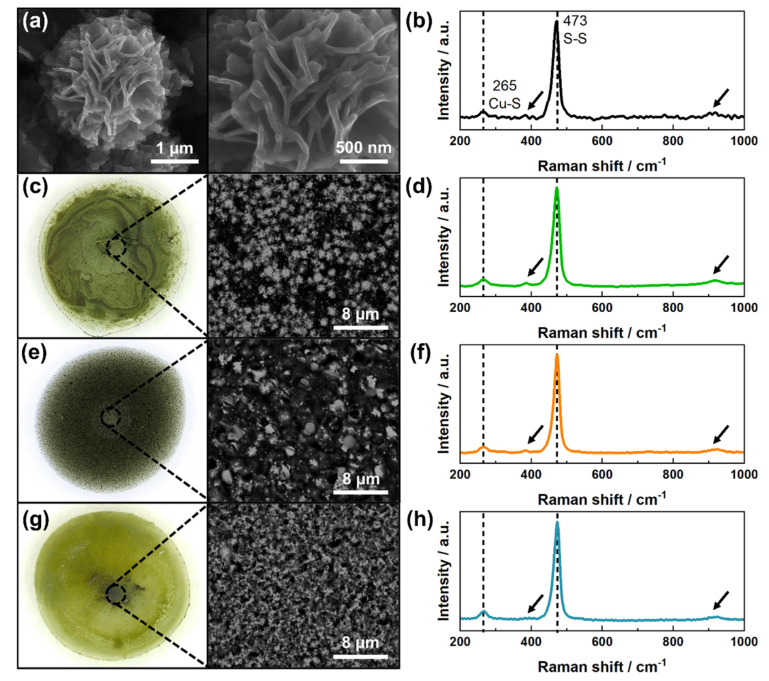
SEM images (**a**,**c**,**e**,**g**) and Raman spectra (**b**,**d**,**f**,**h**) of obtained copper sulfides (**a**,**b**) and Cu-CH (**c**,**d**), Cu-NAF (**e**,**f**), Cu-PVP (**g**,**h**) drop-casted layers. The arrows indicate the characteristic vibration modes for PVP-caped particles.

As can be seen in Figure 1a, the synthesized copper sulfides possessed a flower-like morphology.

Analysis of the SEM images using ImageJ software revealed that they were composed of intersecting nanosheets with a thickness between 40 and 70 nm. The obtained XRD pattern (Figure S1) confirmed that the main phase was digenite, Cu_1_._8_S (PDF-00-047-1748), with a minor contribution of covelline, CuS (PDF-00-006-0464). Furthermore, Raman spectroscopy indicated the presence of PVP on the particle’s surface (Figure 1b). In the sample spectrum, in addition to the bands characteristic for copper sulfides (265 and 473 cm^−1^), vibration modes typical for PVP-caped particles were also detected at 382 cm^−1^ and 934 cm^−1^ (reference PVP spectrum is presented in Figure S2). The latter band can be attributed to C-C ring breathing [27]. The specific surface area (*S_BET_*) of the powder was assessed by Brunauer-Emmett-Teller (BET) theory and was equal to 11.3 m^2^/g. Based on this value, the average particle diameter (*d_BET_*) was estimated using Equation (1):

(1) $$d_{BET}=\frac{6000}{S_{BET}\cdot \rho },$$

where *ρ* is the density of the material; in this case, a value for digenite was assumed (*ρ* = 5.82 g/cm^3^). The calculated average diameter of 91 nm suggests significant surface development since the microflower size observed on the SEM images was around 2.5 µm. Additionally, the recorded adsorption-desorption isotherm was of type III (Figure S3), indicating that only macropores were present within the sample [28].

The distribution of particles on the surface of the electrode can have a significant impact on the outcome of the sensor [29]. Therefore, the morphology of the drop-casted layers composed of polymers and copper sulfides (Cu-polymer) was investigated using optical and scanning electron microscopes. As can be seen in Figure 1c,e,g, the most dense and uniform coverage was obtained when using PVP-based suspension. Interestingly, this polymer was actually not remaining on the electrode surface, but dissolved almost completely when the electrodes were pre-washed, leaving a thin layer of particles firmly attached to the GCE. On the other hand, Nafion and chitosan formed insoluble coatings in neutral and alkaline solutions in which copper sulfides were embedded. Due to the depleted stabilization of the Cu-CH suspension, microflowers were agglomerated and accumulated at the perimeter as well as in the middle of the deposit. For Cu-NAF, the particles were bridged by the polymer and therefore formed small clusters randomly distributed on the electrode surface. Raman spectra (Figure 1d,f,h) revealed that, apart from adsorbed PVP during the synthesis process, no other polymers were present on the copper sulfide surface.

### 3.2. Electrochemical Characterization of Modified Electrodes

**Figure 2:**
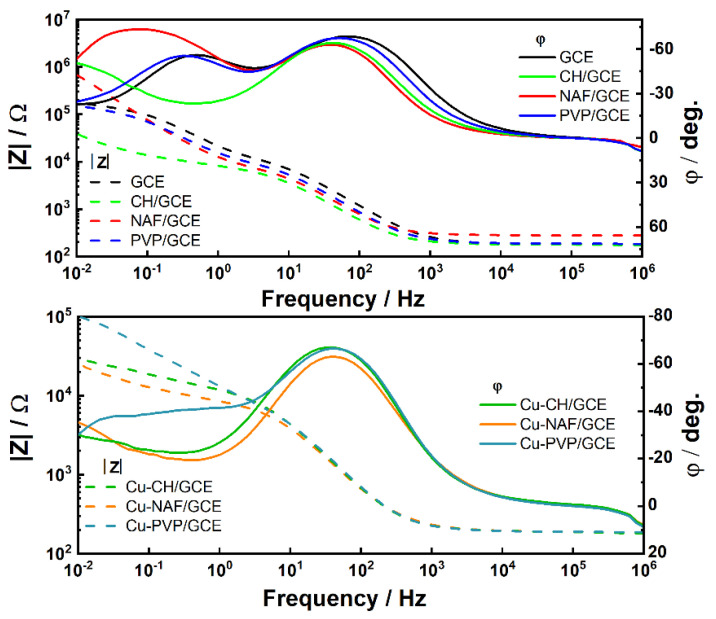
Impedance spectra in the Body configuration for bare and modified GCEs in the 0.1 M KCl + 0.2 mM [Fe(CN)_6_]^3−/4−^ solution.

Electrochemical impedance spectroscopy belongs to the group of techniques that are sensitive to changes occurring at the electrode surface. It is also believed that this method can be applied to determine the direct oxidation of glucose at the electrode, which can be used to study the biosensor response [15]. The impedance spectra for the GCE after different steps of surface modification, i.e., coated with polymer (polymer/GCEs) and modified with nanostructured copper sulfides dispersed in polymer solutions (Cu-polymer/GCEs), were studied. The functionalized electrodes were characterized in an electrochemical cell containing 0.1 M KCl as the supporting electrolyte and 0.2 mM [Fe(CN)_6_]^3−/4−^ as a redox probe couple. In addition, bare GCE and Cu-polymer/GCEs were investigated in 0.1 M NaOH and 0.1 M NaOH + 1 mM glucose electrolytes. The impedance spectra in different representations were analyzed. The absolute value of the impedance *|Z|* and phase angle *φ* versus frequency (Bode plots) are presented in Figure 2.

A wide frequency range was necessary to create a suitable model of the electric circuit. It is well-known that various phenomena are responsible for the shape of the impedance spectrum depending on the frequency range. Due to the complex electrode structure, processes with different relaxation times were involved. Therefore, to match the EIS data, CPE and resistor connected in parallel, as well as a parallel circuit of CPE and connected in series Warburg element with resistor were used. At high frequencies, the impedance is strongly related to characteristics of the electrolyte in contrast to the low-frequency range, where the influence of diffusional mechanisms should be considered. The greatest polymer on the impedance spectrum of the electrode was observed for chitosan. On the other hand, the use of nonionic polyvinylpyrrolidone (PVP) caused an increase in *|Z|* in the low-frequency range.

**Figure 3:**
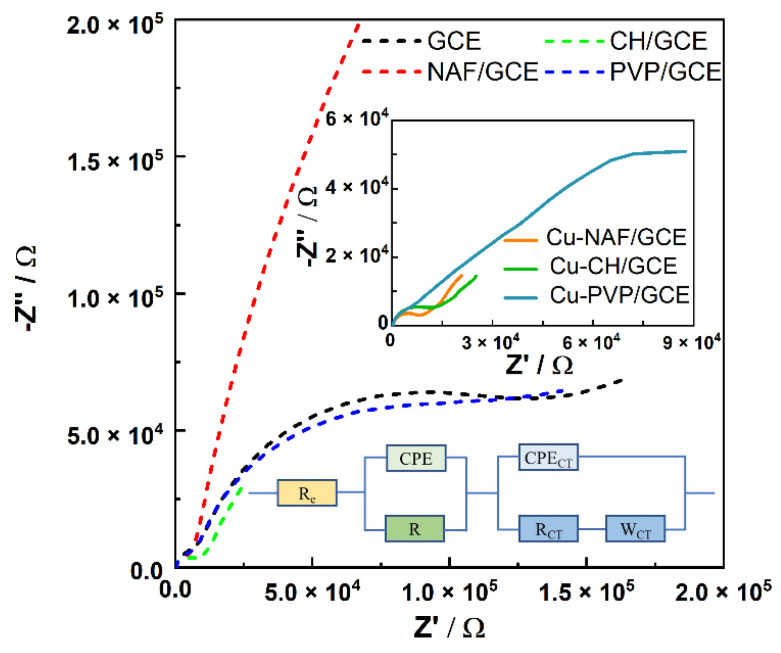
Impedance spectra in the Nyquist configuration for bare and modified GCEs in the 0.1 M KCl + 0.2 mM [Fe(CN)_6_]^3−/4−^ solution. The inset shows the equivalent circuit.

The Nyquist plots of the GCE-based electrodes show the imaginary part Z′′ as a function of the real part Z′ (Figure 3).

The scheme of the fitted equivalent circuit (inset of Figure 3) consists of the electrolyte resistance (R_e_) and two loops connected in series. The first loop composed of R and CPE in parallel can be assigned to the bulk material impedance of polymer/GCE. Electrode processes controlled by diffusional mechanisms are presented as a parallel connection of CPE_CT_ and Faradic impedance [23]. Faradic impedance can be expressed as a series combination of the charge transfer resistance (R_CT_) and the Warburg element (W_CT_). This part of the arrangement is represented by a unity-sloped (45°) straight line at the low-frequency part of Nyquist plots.

Based on the analysis of the impedance spectra, the equivalent circuit parameters, such as charge transfer resistance (*R_CT_*), Warburg impedance modulus (*W_CT_*), and differential capacity of the double layer (*CPE_CT_*), were evaluated and are listed in Table 2.

The constant phase element was defined as:

(2) $$Z_{CPE}=A^{-1}{\left(i\omega \right)}^{-m},$$

where *A* and *m* are constants, *i* is the imaginary unit, and ω represents the angular frequency. The coefficient *m* different from 1 corresponds to a certain deviation from capacitive behavior. For all electrodes studied in this work, the parameter *m* of the CPE_CT_ element takes values close to 0.9–1.0, which corresponds to the Debye capacitor.

The heterogenic rate constant of the electrode reaction *k* was determined using Equation (3) [20]:

(3) $$k=\frac{W_{CT}{\left(D_{O/R}\right)}^{1/2}}{\sqrt{2}R_{CT}},$$

where *D_O/R_* is the diffusion coefficient for [Fe(CN)_6_]^3−/4−^ redox species (*D_O/R_* = 7.2 × 10^−6^ cm^2^/s).

Modification of GCE with chitosan and Nafion resulted in a drop in the charge transfer resistance. The slightly elevated *R_CT_* value for PVP/GCE than for bare GCE can be explained by the formation of a dielectric shell [30], which partially dissolved during the pre-washing step. For all copper sulfide functionalized electrodes, the charge transfer resistance decreased, taking the lowest value for Cu-PVP/GCE. Bare GCE and polymer-coated electrodes exhibited high Warburg moduli, which were lowered after modification with Cu_1.8_S/CuS. The effective reduction of the charge transfer resistance for Cu-PVP/GCE and comparable *W_CT_* values for all electrodes based on copper sulfides resulted in an increased rate constant for this electrode compared to the others.

**Figure 4:**
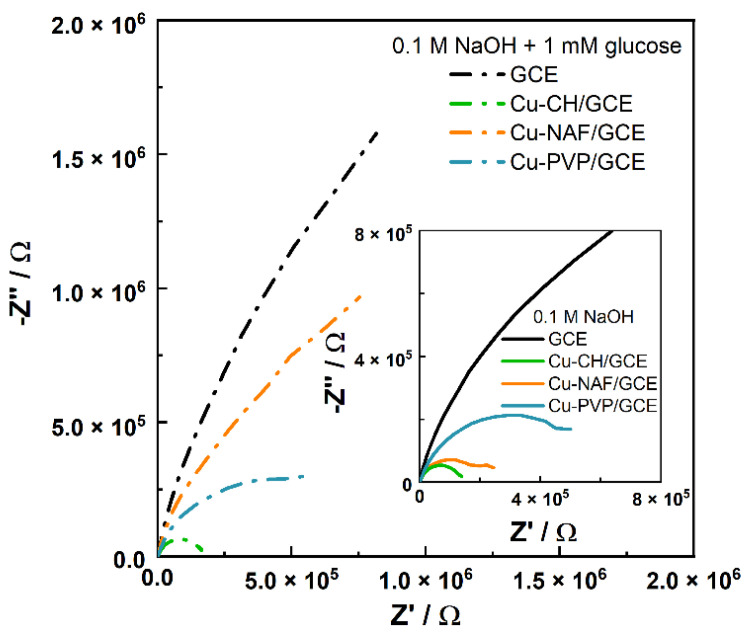
Impedance spectra in the Nyquist configuration for modified GCEs in the 0.1 M NaOH and 0.1 M NaOH + 1 mM glucose solutions.

Impedance spectra of bare and modified GCEs in 0.1 M NaOH solution without and with 1 mM glucose addition are shown in Figure 4.

The Nyquist spectra of Cu-polymer/GCEs take the form of a compressed semicircle in the low-frequency region. The diameter of the semicircle was strongly dependent on the composition of the solution and the design of the electrode. Moreover, it was also related to the active charge transfer resistance of the electrode surface area. As can be seen in Figure 4, the value of the real impedance part increases when glucose is added to the sodium hydroxide solution, which is shown as an increase in the diameter of the semicircles. These apparent changes in the spectra indicate the potential of such an approach for the investigation of glucose adsorption on the electrode surface as well as processes occurring during the electrooxidation process.

The Randles-Sevcik equation was applied for the determination of the electrochemically active surface area (EASA) using cyclic voltammetry in 0.1 M KCl + 1 mM [Fe(CN)_6_]^3−^:

(4) $$I=2.69\cdot 10^{5}\cdot EASA\cdot D_{O/R}\cdot n^{\frac{3}{2}}\cdot C_{0}\cdot v^{\frac{1}{2}},$$

where *I* is the peak current [A], *D_O/R_*—diffusion coefficient of [Fe(CN)_6_]^3−/4−^—redox species (*D_O/R_* = 7.2 × 10^−6^ cm^2^ /s), *n*—number of electrons involved in the redox reaction (*n* = 1), *C_0_*—concentration of redox species (*C_0_* = 1 mM), and *v*—scan rate [mV/s].

Analysis of the results indicated that the highest EASA of 50.98 mm^2^ possessed chitosan-modified GCE (Figure S4a). This may be explained by the fast transport of ferrocyanide anions through chitosan and their incorporation into the film (the modifying layer became yellow after the experiment), as also observed by Cruz et al. and Martinez-Huitle et al. [31,32]. On the other hand, for NAF/GCE, the active surface area could not be determined using this approach due to the fact that this anionic polymer repelled negatively charged [Fe(CN)_6_]^3−^ ions. Therefore, no explicit redox peak was observed (Figure S4b). These observations suggest that obtained values should be interpreted in the view of the applied particular redox probe [33]. In the case of the PVP-based electrode, its surface was close to that of the bare GCE (4.22 and 3.46 mm^2^, respectively). The lower EASA of bare GCE than its geometric area (7.07 mm^2^) can be attributed to the applied activation procedure and changes introduced in the surface composition and roughness [34,35]. The amperometric response of the electrodes during the oxidation and reduction process, as well as the influence of electrodes activation on the voltammograms, are presented in Figure S5. Among the GCEs modified with copper sulfides, the highest EASA was obtained for Cu-PVP/GCE (8.63 mm^2^), while the calculated values for Cu-NAF/GCE and Cu-CH/GCE were comparable (2.82 and 1.79 mm^2^, respectively). This was attributed to the most uniform coverage with particles of the substrate electrode ensured by PVP, which provided steric stabilization of copper sulfides within the prepared suspension and drying droplet. Details on stabilization mechanisms provided by different polymers have previously been reported [24].

### 3.3. Glucose Detection

**Figure 5:**
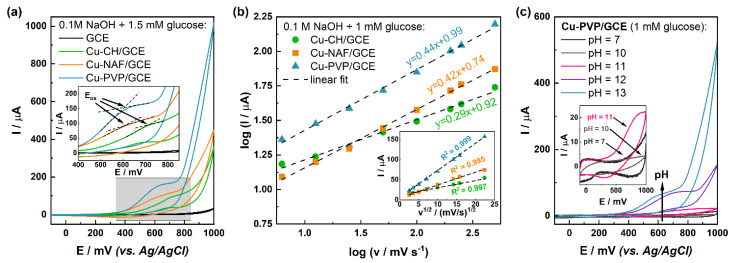
Comparison of: (**a**) cyclic voltammograms for bare and modified GCEs in the presence of 1.5 mM glucose at a scan rate of 100 mV/s (inset shows the magnified potential range of glucose oxidation peak formation), (**b**) logarithmic dependency of scan rate on generated peak current (inset shows dependency of peak current on the scan rate) and (**c**) cyclic voltammograms for Cu-PVP/GCE in the presence of 1 mM glucose in electrolytes with different pH values at a scan rate of 100 mV/s (inset shows magnified region of lower current values).

The first step in evaluating the glucose-sensing abilities of the modified electrodes involved the determination of the glucose oxidation potential by means of CV. For this purpose, cyclic voltammograms were recorded after subsequent glucose addition in the concentration range 0–1.5 mM. As can be seen in Figure 5a, the highest peak current (1.5 mM glucose) was obtained for Cu-PVP/GCE, whereas Cu-NAF/GCE and Cu-CH/GCE generated visibly smaller signals.

Moreover, for the latter, also a shift in the position of the oxidation peak (*E_ox_* = 680 mV) compared to other electrodes (*E_ox_* = 590 mV) was detected. These observations are in good agreement with results from EIS, which indicated preferable electrochemical properties of Cu-PVP/GCE (low charge transfer resistance and high heterogeneous rate constant of the electrode reaction) over chitosan- and Nafion-based electrodes.

To determine whether glucose oxidation by modified electrodes is a diffusion- or surface-controlled process, voltammograms were recorded at different scan rates in the electrolyte containing 1 mM glucose. Subsequently, the logarithmic dependence of the scan rate on the generated peak current was plotted (Figure 5b). The obtained slope should take values between 0.5 and 1, indicating diffusion limitation or surface control, respectively [36]. It was found that the slopes in the cases of Cu-PVP/GCE and Cu-NAF/GCE were 0.44 (R^2^ = 0.996) and 0.42 (R^2^ = 0.997), respectively, which is relatively close to the expected theoretical value for the diffusion-controlled process (0.5). The slope for Cu-CH/GCE was almost two times lower (0.29, R^2^ = 0.988). This may be explained by the characteristic sigmoidal shape of the voltammogram for this electrode, indicating the occurrence of a multiplied microelectrode effect [37]. Formed on the GC macroelectrode, the separated domains of particles (due to instability of casted suspension) served as individual active centers. Similar conclusions were drawn based on plots of peak current versus the square root of the scan rate. The fitted regression lines possessed very high correlation coefficients, further proving the diffusion-controlled process [38]. This mechanism of glucose oxidation was also reported by Wei et al. [39]. On the other hand, on electrodes modified with cobaltosic oxide (Co_3_O_4_) developed by Ding et al., a surface-limited detection process occurred [40]. Furthermore, in the case of carbon fibers coated with CuS proposed by Keerthi et al. [41], electrooxidation was determined by surface reactions.

Another investigated issue was the influence of the electrolyte pH on the signal obtained from the glucose. In most articles devoted to non-enzymatic glucose sensors based on copper sulfides, an alkaline medium was used for the measurements (Table S1). This is due to the expected significant influence of the premonolayer of OH_ads_ suggested by the Incipient Hydrous Oxide-Adatom Mediator (IHOAM) theory [8]. It was originally proposed by Burke for the explanation of the electrocatalytic activity of noble metals in an alkaline medium. However, several articles can also be found in which the reported electrodes worked in PBS (pH = 7), without apparent alterations in the modification procedure or the measurement methodology. To investigate this issue, we recorded voltammograms for Cu-PVP/GCE after the subsequent addition of glucose to electrolytes (0.1 M PBS and diluted 0.1 M NaOH) with adjusted pH values (7, 10, 11, 12, and 13). As can be seen in Figure 5c and S6, below pH = 12, almost no signal was generated when glucose was added. A further increase in the pH value led to a slight decrease in the peak current from the value of 73.42 to 63.51 µA (for pH = 12 and pH = 13, respectively), as well as a significant shift of the oxidation potential from 700 to 590 mV. This underlines the role of hydroxyl ions in the process of glucose electrocatalytic oxidation by metal sulfides. Similar observations were reported in the article by Keerthi et al., in which a screen-printed carbon electrode modified with CuS-decorated carbon nanofibers did not work at neutral pH, whereas it exhibited excellent sensing abilities in 0.1 M NaOH [41]. In another study, Fu et al. investigated the influence of electrolyte pH (11.5, 11.9, 12.3, 12.7, and 13.0) on the generated current density (*j*) for core-shell CuS-Cu_2_S decorated carbon nanotube-graphene composites [42]. It was found that the maximum value of *j* was obtained at pH = 12.7, while a further increase of pH resulted in a signal drop, which is in agreement with our results. Moreover, the same behavior was observed in the case of electrodes working at neutral pH developed by Gao et al. [26]. In their research article, MoS_2_-CuS/GCE electrodes were tested in PBS with different pH values ranging from 5.5 to 8.0. The highest peak current was obtained at pH = 7.2.

Based on the findings of Burke, subsequent studies on glucose oxidation via transition metals [8,9,10,43,44,45], and our results, the following electrocatalytic mechanism occurring on the proposed Cu_1.8_S/CuS modified electrodes was adapted:

(5) $${\mathrm{Cu}}^{2+}\to {\mathrm{Cu}}^{3+}+e^{-}$$

(6) $$\mathrm{CuS}+{\mathrm{OH}}^{-}\to \mathrm{CuSOH}+e^{-}$$

(7) $$2\mathrm{CuSOH}+2e^{-}+C_{6}H_{12}O_{6}\to 2\mathrm{CuS}+C_{6}H_{10}O_{6}+2H_{2}O$$

Glucose detection was performed by chronoamperometry. Appropriate calibration curves were prepared in the range of 0–1 mM (step 0.1 mM). The limits of detection (LOD) and quantification (LOQ) were evaluated using the signal-to-noise (S/N) method with S/N ratios of 3 and 10, respectively. Sensitivity was calculated based on the determined slopes and EASA. The assessed values are listed in Table 3.

All samples exhibited comparable sensitivity; however, generated signals differed significantly. This may be attributed to the available active sites of copper sulfides. In the case of chitosan- and Nafion-based electrodes, these polymers blocked some of them, as also indicated by decreased values of EASA and higher Warburg impedance modulus [14,46]. The small background current reducing LOD and LOQ for Cu-PVP/GCE may be explained by its lower *CPE_CT_* compared to that of other electrodes [20].

**Figure 6:**
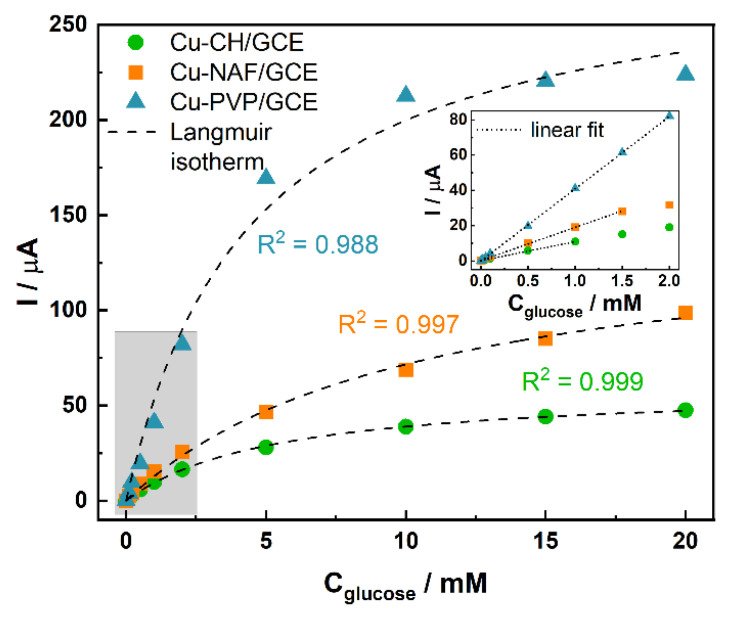
Amperometric response of modified electrodes to increasing glucose concentrations with fitted Langmuir isotherms. The inset shows the linear response range of the electrodes with fitted regression lines.

In this study, emphasis was placed on the evaluation of the generated current (*I*) values over a wide range of concentrations (*C_glucose_* = 0.01–20 mM). It was found that in the very limited range, the current dependency on *C_glucose_* is linear, whereas altogether, it follows a Langmuir isotherm (Figure 6).

This is in agreement with the study by Ding et al. [40], in which the following equation was proposed:

(8) $$I=\frac{K_{A}K_{B}C_{t}C_{glucose}}{1+K_{A}C_{glucose}}=\frac{KC_{glucose}}{1+K_{A}C_{glucose}},$$

where *K_A_* is an adsorption equilibrium constant, *K_B_*—rate constant, *C_t_*—total molar concentration of active sites on the materials surface, and *K*—equivalent constant.

The determined values of *K* and *K_a_,* as well as the correlation coefficients (R^2^) of the proposed fittings, are summarized in Table 3. The results indicated that the model matched very well with our data (Figure 6). Moreover, in the case of a low concentration range (*K_a_C_glucose_ << 1*), Equation (8) can be simplified to *I = KC_glucose_*, which is in good agreement with the evaluated slopes of the calibration curves. Ding et al. related the applicability of this approach to surface catalytic reactions [40]. The observed gradual depletion of the generated current may be explained by the irreversible character of the electrooxidation process [47]. The linear range of the electrode response (R^2^ > 0.999) was evaluated to be 0.06–1.00 mM for Cu-CH/GCE, 0.06–1.50 mM for Cu-NAF/GCE, and 0.02–2.00 mM for Cu-PVP/GCE.

Additionally, the repeatability and reproducibility of the investigated electrodes were evaluated. The repeatability test consisted of comparing the responses of the same electrode to 0.5 mM glucose addition during three independent measurements. The calculated relative standard errors (RSE) were equal to 4.4%, 3.1%, and 3.2% for Cu-CH/GCE, Cu-NAF/GCE, and Cu-PVP/GCE, respectively, indicating satisfactory repeatability. To determine reproducibility, three different electrodes were fabricated, and calibration curves in the glucose concentration range 0–1 mM were prepared using them (Figure S7). The RSE values of the calibration slopes were acceptably low for Cu-NAF/GCE and Cu-PVP/GCE (4.3% and 3.9%, respectively). However, the Cu-CH/GCEs exhibited a high deviation of the generated signal (RSE = 25.8%). This may be attributed to the unstable drop-casted suspension based on chitosan and, therefore, the variable arrangement of particles in dry deposits [24].

## 4. Conclusions

This work underlines crucial steps that should be undertaken when evaluating the impact of the single working electrode component on the sensor analytical performance. We proposed a double-track approach. The first considered aspect included evaluation of the morphology and structure of the modifying layers, with special emphasis on the distribution of particles on the surface. Another important issue was the determination of changes brought by each modifying component to the electrode electrochemical properties, which was achieved efficiently by utilizing electrochemical impedance spectroscopy. The information provided by this technique combined with microscope observations allowed us to predict the analytical performance of modified electrodes. In our case, the application of PVP as a binder in drop-casted suspension ensured both uniform coverage of the GCE surface with copper sulfides and superior electrochemical properties in comparison with chitosan- and Nafion-based electrodes. We also conducted comprehensive studies on the influence of electrolyte pH on the Cu-PVP/GCE response using cyclic voltammetry. This technique also served to determine the limiting process during detection. Nonetheless, further extension of the EIS measurements is required to gain a better insight into the electrocatalytic activity of copper sulfides in glucose oxidation. This includes measurements in electrolytes covering a wider range of pH values and containing different glucose additions, as well as conducting experiments in a three-electrode system at the applied potential.

## Figures and Tables

**Table 1:** Composition of drop-casted suspensions.

Drop-Casted Suspension	Binder	Binder wt.%	Solvent	Cu_1_._8_S/CuS wt.%
CH	chitosan	0.5	25% CH_3_COOH	-
NAF	Nafion	0.5	water/1-propanol	-
PVP	PVP	1.0	25% CH_3_COOH	-
Cu-CH	chitosan	0.5	25% CH_3_COOH	0.25
Cu-NAF	Nafion	0.5	water/1-propanol	0.25
Cu-PVP	PVP	1.0	25% CH_3_COOH	0.25

**Table 2:** Summary of equivalent circuit parameters and heterogenic rate constants of the electrode reaction evaluated based on EIS measurements.

Modified Electrode	*R_CT_* [kΩ]	*W_CT_* [kΩ/s^1/2^]	*CPE_CT_* [μF]	*k* × 10^−5^ [m/s]
GCE	111.15	236.68	4.055	4.61
CH/GCE	9.43	269.71	9.628	54.36
Cu-CH/GCE	4.67	49.69	45.262	20.21
NAF/GCE	5.75	108.14	2.997	35.75
Cu-NAF/GCE	8.03	50.19	113.810	11.88
PVP/GCE	119.65	106.49	17.710	1.69
Cu-PVP/GCE	2.81	35.29	2.438	23.87

**Table 3:** Analytical performance of the electrodes.

Electrode	Slope [µA/mM]	LOD [mM]	LOQ [mM]	Sensitivity [µA/mM·cm^2^]	*K*	*K_a_*	R^2^
Cu-CH/GCE	14.2 ± 0.4	0.016	0.054	793.3	11.128	0.186	0.999
Cu-NAFGCE	22.4 ± 0.8	0.017	0.055	794.3	14.057	0.096	0.997
Cu-PVP/GCE	68.6 ± 1.0	0.006	0.019	794.7	65.819	0.227	0.988

## Data Availability

Not applicable.

## Supplementary Materials

The following are available online at https://www.mdpi.com/article/10.3390/s21237928/s1, Figure S1. XRD pattern of the obtained copper sulfides; Figure S2. Raman spectra of the PVP powder; Figure S3. BET analysis: N_2_ adsorption-desorption isotherms for copper sulfides; Figure S4. Dependency of the generated peak current on the square root of the scan rate for bare and modified electrodes with fitted regression lines (a) and voltammograms recorded with different scan rates (6.25–500 mV) for NAF/GCE; Figure S5. The activation procedure: (a) oxidation (30 s) and subsequent reduction (10 s), and (b) its influence on the voltammograms; Figure S6. Voltammograms recorded for Cu-PVP/GCE in electrolytes with different pH after subsequent additions of glucose in the concentration range of 0–1.2 mM (scan speed: 100 mV/s); Figure S7. Calibration curves in the glucose concentration range of 0–1 mM for three different electrodes: (a) Cu-CH/GCE, (b) Cu-NAF/GCE, and (c) Cu-PVP/GCE; Table S1: Summary of the reported glucose sensors based on copper sulfides with special reference to used electrolyte during the measurements. References [26,27,39,42,43,44,48,49,50,51,52,53,54,55,56,57,58,59,60,61] are cited in the supplementary materials.
