# Supplementary material for: Electrochemical Characterization of Modified Glassy Carbon Electrodes for Non-Enzymatic Glucose Sensors
Source: Sensors (Basel). 2021 Nov 27;21(23):7928. doi: 10.3390/s21237928 (PMC8659783; doi:10.3390/s21237928)
Supplement: Supplementary file 1 [file sensors-21-07928-s001.zip › sensors-1460058-supplementary.pdf]

Supplementary Materials

Electrochemical Characterization of Modified Glassy Carbon Electrodes for Non-Enzymatic Glucose Sensors

Julia Maria Mazurków *, Anna Kusior and Marta Radecka

Faculty of Materials Science and Ceramics, AGH University of Science and Technology, al. Mickiewicza 30, 30-059 Kraków, Poland; akusior@agh.edu.pl (A.K.); radecka@agh.edu.pl (M.R.)

* Correspondence: mazurkow@agh.edu.pl; Tel.: +48-12-617-24-68

Number of Pages: 7

Number of Figures: 7

Number of Tables: 1

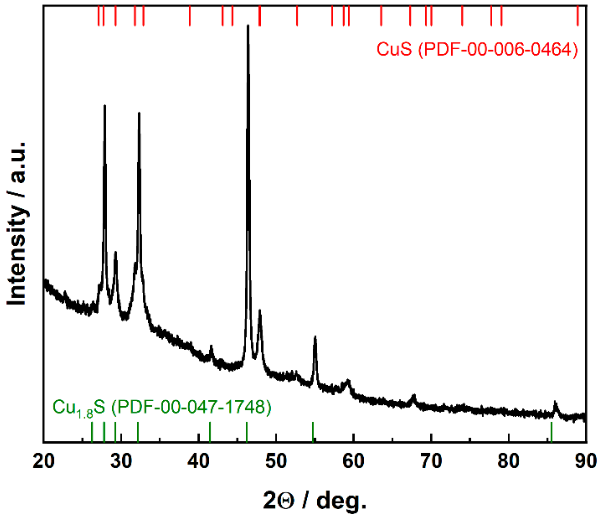

Figure S1. XRD pattern of the obtained copper sulfides.

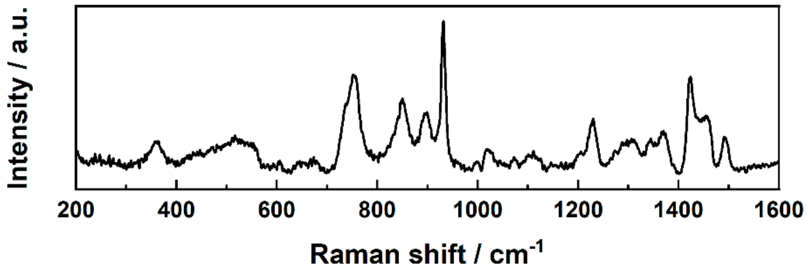

Figure S2. Raman spectra of the PVP powder.

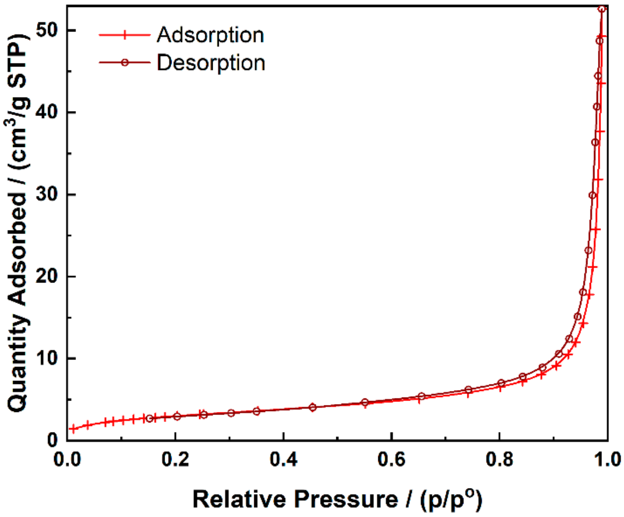

Figure S3. BET analysis: N_2 adsorption-desorption isotherms for copper sulfides.

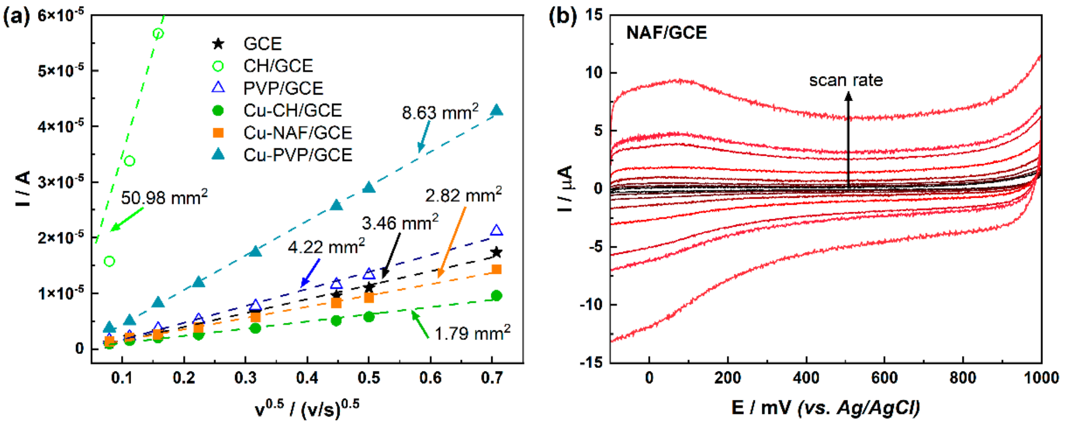

Figure S4. Dependency of the generated peak current on the square root of the scan rate for bare and modified electrodes with fitted regression lines **(a)** and voltammograms recorded with different scan rates (6.25–500 mV) for NAF/GCE **(b)**.

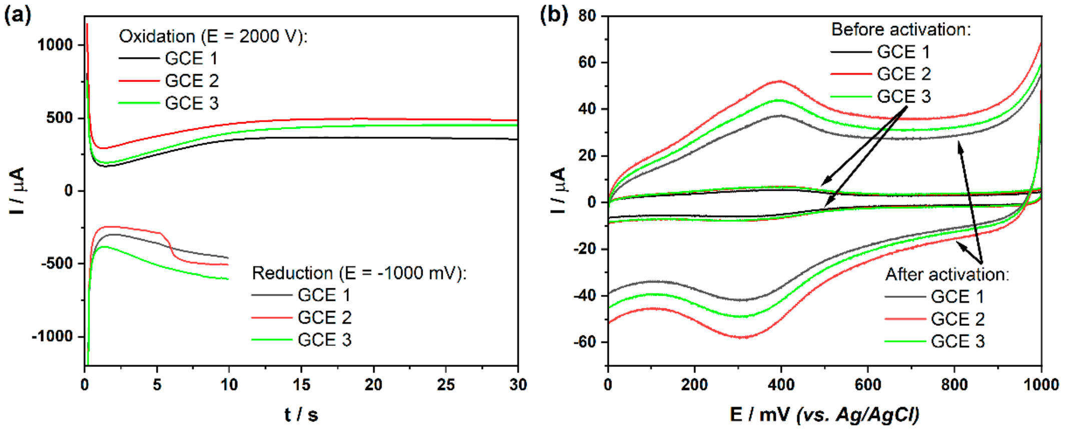

Figure S5. The activation procedure: **(a)** oxidation (30 s) and subsequent reduction (10 s), and **(b)** its influence on the voltammograms.

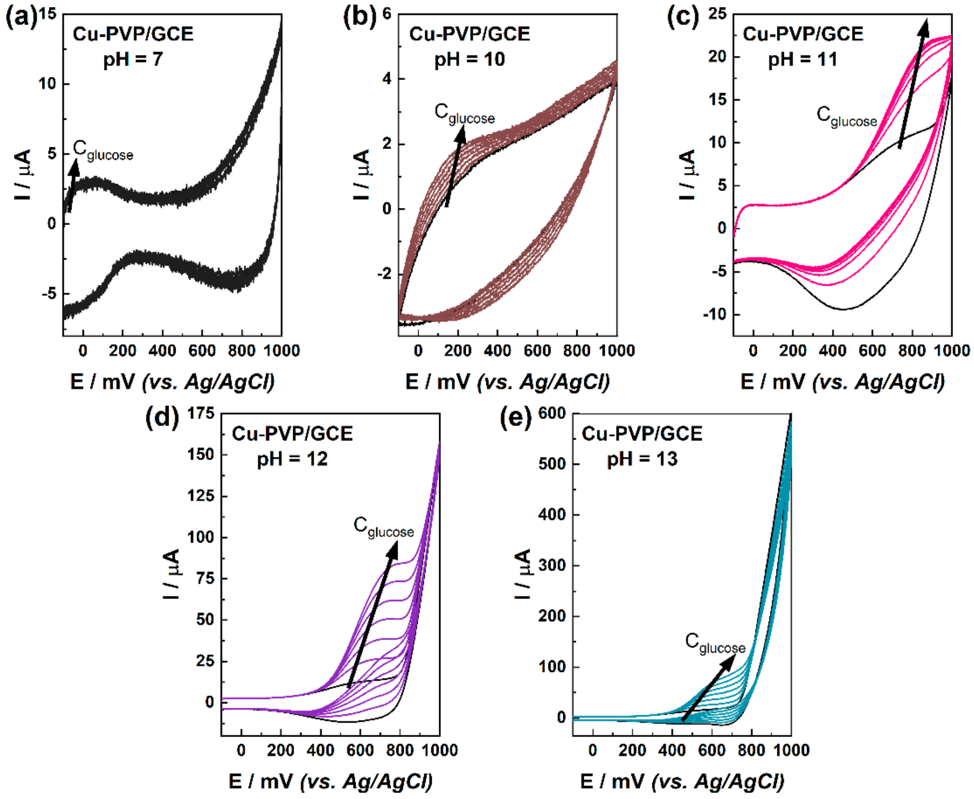

Figure S6. Voltammograms recorded for Cu-PVP/GCE in electrolytes with different pH after a subsequent addition of glucose in the concentration range of 0–1.2 mM (scan speed: 100 mV/s).

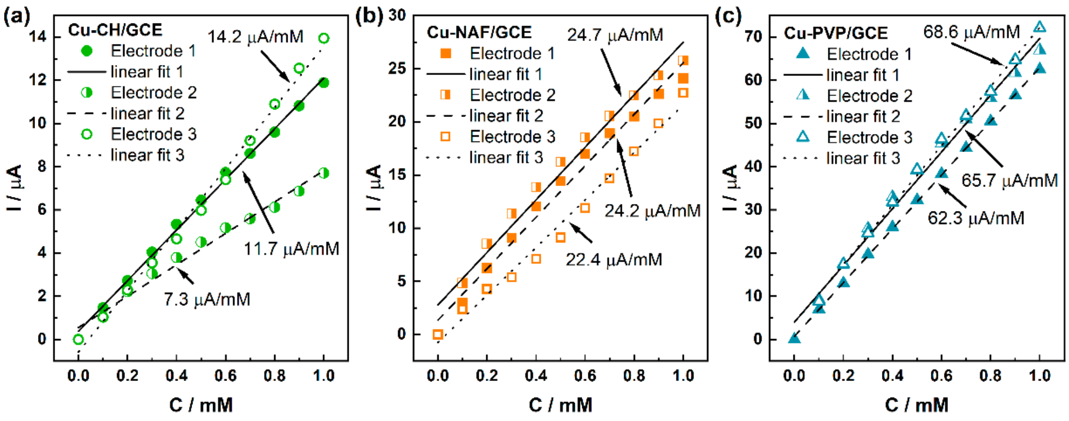

Figure S7. Calibration curves in the glucose concentration range of 0–1 mM for three different electrodes: **(a)** Cu-CH/GCE, **(b)** Cu-NAF/GCE, and **(c)** Cu-PVP/GCE. Values of calibration slopes are given in the graphs.

Table S1. Summary of the reported electrochemical glucose sensors based on copper sulfides with special reference to used electrolyte during the measurements.

**Modified Electrode (Binder)**	**Electrolyte**	**Applied Potential [mV] / Reference Electrode**	**Sensitivity [μA/(mM·cm²)]**	**Reference**
CuS/GCE (Nafion)	**PBS (pH = 9.2)**	200 / SCE (saturated)	7842 [μA/mM]	[1]
Cu_2S-DWCNTs/GCE, Cu_2S-MWCNTs/GCE (Nafion)	**10 mM PBS (pH = 7.2)**	500 / Ag/AgCl (3 M KCl)	35, 5.0	[2]
CuS/GCE (Nafion)	**0.1 M Na_2HPO_4**	CV / SCE (saturated)	117.3	[3]
CuS/GCE (chitosan)	**0.1 M PBS (pH = 7.2)**	CV / SCE (saturated)	5.86 [μA/mM]	[4]
CuS/GCE (-)	0.1 M NaOH	700 / SCE (saturated)	64.97	[5]
Cu_2S/Cu foam (-)	0.1 M NaOH	450 / SCE (saturated)	11750.8	[6]
Cu_xO/Cu_2S/GCE (Nafion)	0.01 M NaOH	450 / SCE (saturated	2688	[7]
CuS/GCE (Nafion)	0.1 M NaOH	500 / Ag/AgCl (3M KCl)	1007	[8]
CuS/GCE (PVB)	**0.1 M PBS (pH = 5.5–8.0)**	180 / Ag/AgCl (3.5 M KCl)	-	[9]
CuS/Ni foam (-)	0.5 M NaOH	550 / Ag/AgCl (saturated)	8337	[10]
S-rGO/CuS/GCE, S-rGO/CuS/RDE (-)	0.1 M NaOH	480 / Ag/AgCl (saturated)	- 429.4	[11]
rGO/CuS/GCE (-)	0.1 M NaOH	400 / SCE (saturated)	53.5	[12]
$Cu_2O@Cu_{1.8}S$/RDE (Nafion)	0. 1 M NaOH	500 / Ag/AgCl (saturated)	3630	[13]
CuS/XGCNFs/SPE (-)	0.1 M NaOH, **PBS (pH = 7)**	570 / Ag/AgCl (-)	23690, not working	[14]
CuS/Cu_2S-GO-MWCNTs/GCE (Nafion)	0.1 M NaOH (pH = 11.5, 11.9, 12.3, 12.7, 13.0)	600 / Ag/AgCl (-)	1923 (pH = 12.7)	[15]
Cu_2S/GCE, CuS/GCE, Cu_7S_4/GCE (Nafion)	0.1 M NaOH	500 / Ag/AgCl (saturated)	3610.1, 3594.9, 3728.7	[16]
CuS/GCE	0.1 M NaOH	500 / no information	7.18 [μA/mM]	[17]
CuS/Cu_2O/CuO/Cu (-)	0.1 M NaOH	600 / Ag/AgCl (saturated)	4262	[18]
Cu_2S/ABA/GCE (-)	**1 mM PBS (pH = 7.4)**	1000 / SCE (saturated)	38210	[19]
CuS/GCE, CuS/Cu_2S/GCE (-)	0.1 M NaOH	600 / SCE (saturated)	130.2, 321.3	[20]
Cu_2S-Cu_2O/GCE (Nafion)	0. 1 M NaOH	650 / Ag/AgCl (saturated)	1876	[21]

Abbreviations: GCE – glassy carbon electrode, DWCNTs – double-walled carbon nanotubes, MWCNTs – multi-walled carbon nanotubes, CV – cyclic voltammetry glucose detection, PVB – poly(vinyl butyral), RDE – rotating disk electrode, GO – graphene oxide, rGO – reduced graphene oxide, S-rGO – sulfur doped reduced graphene oxide, XGCNFs – xanthan gum carbon nanofibers, SPE – screen printed electrode, ABA – m-aminobenzoic acid.

References

1. Zhang, X.; Wang, G.; Gu, A.; Wei, Y.; Fang, B. CuS nanotubes for ultrasensitive nonenzymatic glucose sensors. *Chem. Commun.* **2008**, 5945–5947, doi:10.1039/b814725f.
2. Myung, Y.; Jang, D.M.; Cho, Y.J.; Kim, H.S.; Park, J.; Kim, J.U.; Choi, Y.; Lee, C.J. Nonenzymatic amperometric glucose sensing of platinum, copper sulfide, and tin oxide nanoparticle-carbon nanotube hybrid nanostructures. *J. Phys. Chem. C* **2009**, *113*, 1251–1259, doi:10.1021/jp806633j.
3. Lin, J.; Tao, F.; Wang, L.; Chen, L.; Ying, Y.; Zhang, L.; Liu, H.; Xia, M. Solvothermal synthesis of sphere-like CuS microcrystals and improvement as nonenzymatic glucose sensor. *J. Mater. Sci.* **2013**, *48*, 5509–5516, doi:10.1007/s10853-013-7345-2.
4. Yang, Y.J.; Zi, J.; Li, W. Enzyme-free sensing of hydrogen peroxide and glucose at a CuS nanoflowers modified glassy carbon electrode. *Electrochim. Acta* **2014**, *115*, 126–130, doi:10.1016/j.electacta.2013.10.168.
5. Liu, X.; Ai, L.; Jiang, J. Interconnected porous hollow CuS microspheres derived from metal-organic frameworks for efficient adsorption and electrochemical biosensing. *Powder Technol.* **2015**, *283*, 539–548, doi:10.1016/j.powtec.2015.06.016.
6. Lu, W.; Sun, Y.; Dai, H.; Ni, P.; Jiang, S.; Wang, Y.; Li, Z.; Li, Z. Fabrication of cuprous sulfide nanorods supported on copper foam for nonenzymatic amperometric determination of glucose and hydrogen peroxide. *RSC Adv.* **2016**, *6*, 90732–90738, doi:10.1039/c6ra18641f.
7. Xu, X.; Jin, H.; Ren, Q.; Liu, A.; Li, J.; Yin, D.; Feng, X.; Dong, X.; Wang, J.; Wang, S. Electrochemical synthesis of Cu_xO/Cu_2S nanocomposites as nonenzymatic glucose sensor. *Int. J. Electrochem. Sci.* **2016**, *14*, 5637–5645, doi:10.20964/2019.06.38.
8. Radhakrishnan, S.; Kim, H.Y.; Kim, B.S. A novel CuS microflower superstructure based sensitive and selective nonenzymatic glucose detection. *Sensors Actuators, B Chem.* **2016**, *233*, 93–99, doi:10.1016/j.snb.2016.04.056.
9. Gao, Z.; Lin, Y.; He, Y.; Tang, D. Enzyme-free amperometric glucose sensor using a glassy carbon electrode modified with poly(vinyl butyral) incorporating a hybrid nanostructure composed of molybdenum disulfide and copper sulfide. *Microchim. Acta* **2017**, *184*, 807–814, doi:10.1007/s00604-016-2061-7.
10. Kim, W. Bin; Lee, S.H.; Cho, M.; Lee, Y. Facile and cost-effective CuS dendrite electrode for non-enzymatic glucose sensor. *Sensors Actuators, B Chem.* **2017**, *249*, 161–167, doi:10.1016/j.snb.2017.04.089.
11. Karikalan, N.; Karthik, R.; Chen, S.M.; Karuppiah, C.; Elangovan, A. Sonochemical Synthesis of Sulfur Doped Reduced Graphene Oxide Supported CuS Nanoparticles for the Non-Enzymatic Glucose Sensor Applications. *Sci. Rep.* **2017**, *7*, 1–10, doi:10.1038/s41598-017-02479-5.
12. Yan, X.; Gu, Y.; Li, C.; Zheng, B.; Li, Y.; Zhang, T.; Zhang, Z.; Yang, M. A non-enzymatic glucose sensor based on the CuS nanoflakes-reduced graphene oxide nanocomposite. *Anal. Methods* **2018**, *10*, 381–388, doi:10.1039/c7ay02290e.

13. Cao, M.; Wang, H.; Ji, S.; Zhao, Q.; Pollet, B.G.; Wang, R. Hollow core-shell structured $Cu_2O@Cu_{1.8}S$ spheres as novel electrode for enzyme free glucose sensing. *Mater. Sci. Eng. C* **2019**, *95*, 174–182, doi:10.1016/j.msec.2018.10.082.
14. Keerthi, M.; Mutharani, B.; Chen, S.M.; Ranganathan, P. Carbon fibers coated with urchin-like copper sulfide for nonenzymatic voltammetric sensing of glucose. *Microchim. Acta* **2019**, *186*, doi:10.1007/s00604-019-3915-6.
15. Fu, Y.; Jin, W. Facile synthesis of core-shell CuS-Cu_2S based nanocomposite for the high-performance glucose detection. *Mater. Sci. Eng. C* **2019**, *105*, 110120, doi:10.1016/j.msec.2019.110120.
16. Cao, M.; Wang, H.; Kannan, P.; Ji, S.; Wang, X.; Zhao, Q.; Linkov, V.; Wang, R. Highly efficient non-enzymatic glucose sensor based on Cu_xS hollow nanospheres. *Appl. Surf. Sci.* **2019**, *492*, 407–416, doi:10.1016/j.apsusc.2019.06.248.
17. Zhu, J.; Peng, X.; Nie, W.; Wang, Y.; Gao, J.; Wen, W.; Selvaraj, J.N.; Zhang, X.; Wang, S. Hollow copper sulfide nanocubes as multifunctional nanozymes for colorimetric detection of dopamine and electrochemical detection of glucose. *Biosens. Bioelectron.* **2019**, *141*, 111450, doi:10.1016/j.bios.2019.111450.
18. Wei, C.; Zou, X.; Liu, Q.; Li, S.; Kang, C.; Xiang, W. A highly sensitive non-enzymatic glucose sensor based on CuS nanosheets modified Cu_2O/CuO nanowire arrays. *Electrochim. Acta* **2020**, *334*, 135630, doi:10.1016/j.electacta.2020.135630.
19. Myeni, N.; Perla, V.K.; Ghosh, S.K.; Mallick, K. Organic matrix stabilized copper sulfide nanoparticles: Synthesis, characterization and application in glucose recognition. *Mater. Today Commun.* **2020**, *25*,
20. Huang, W.; Liu, F.; Huang, Y.; Yang, W.; Zhong, H.; Peng, J. Facile one-pot synthesis of hollow-structured CuS/Cu_2S hybrid for enhanced electrochemical determination of glucose. *Electrochemistry* **2021**, *89*, 340–347, doi:10.5796/electrochemistry.21-00027.
21. Zhang, S.; Mou, X.; Cui, Z.; Hou, C.; Yang, W.; Gao, H.; Luo, X. Partial sulfidation for constructing Cu_2O-CuS heterostructures realizing enhanced electrochemical glucose sensing. *New J. Chem.* **2021**, *45*, 7204–7209, doi:10.1039/d1nj00298h.
